# Spatial distribution and functional integration of displaced retinal ganglion cells

**DOI:** 10.1038/s41598-025-91045-5

**Published:** 2025-02-28

**Authors:** Sabrina Duda, Christoph T. Block, Dipti R. Pradhan, Yousef Arzhangnia, Alina Klaiber, Martin Greschner, Christian Puller

**Affiliations:** 1 Visual Neuroscience, Department of Neuroscience, Carl von Ossietzky University, 26111 Oldenburg, Germany; 2 Department of Computational Neuroethology, Max Planck Institute for Neurobiology of Behavior – caesar, Bonn, Germany

**Keywords:** Neuroscience, Retina

## Abstract

The retina contains distinct types of ganglion cells, which form mosaics with cells of each type at each position of the visual field. Displaced retinal ganglion cells (dRGCs) occur with cell bodies in the inner nuclear layer (INL), and regularly placed RGCs with cell bodies in the ganglion cell layer. An example of mammalian dRGCs are M1-type intrinsically photosensitive ganglion cells (ipRGCs). Little is known, however, about their relationship with regularly placed ipRGCs. We identified mouse ipRGC types M1, M2, and M4/sONɑ by immunohistochemistry and light microscopy. Reconstruction of immunolabeled mosaics from M1 and sONɑ RGCs indicated that dRGCs tiled the retina with their regular RGC partners. Multi-electrode array recordings revealed conventional receptive fields of displaced sONɑ RGCs which fit into the mosaic of their regular counterparts. An RGC distribution analysis showed type-specific dRGC patterns which followed neither the global density distribution of all RGCs nor the local densities of corresponding cell types. The displacement of RGC bodies into the INL occurs in a type-dependent manner, where dRGCs are positioned to form complete mosaics with their regular partners. Our data suggest that dRGCs and regular RGCs serve the same functional role within their corresponding population of RGCs.

**Supplementary Information:**

The online version contains supplementary material available at 10.1038/s41598-025-91045-5.

## Introduction

Retinal ganglion cells (RGCs) consist of a heterogeneous group of more than 40 different cell types in mice^1,2^. They can be separated based on the large diversity of morphologies, molecular identities, and light response properties^1–4^. Cell bodies of individual RGC types are regularly spaced and their dendrites are arranged in a mosaic which tiles the retina without leaving gaps^5,6^. Regional specializations of these mosaics exist across the mammalian retina in response to lifestyle and habitat of different species, such as the cat area centralis, the rabbit visual streak, or the human fovea^7–9^. These regions contain increased RGC densities to enhance visual acuity, for instance, in distinct areas of the visual field^10,11^. In mice, RGCs have been thought to have a central to peripheral density gradient^12^. However, recent work showed distinct density gradients for certain types, such as the sustained ON alpha (sONɑ) RGC, contrary to the overall density distribution of RGCs^11,13–16^.

Independent of these specializations, a common feature of all RGC types is their cell body position in the ganglion cell layer (GCL). However, there are poorly understood exceptions to this rule, where cell bodies of displaced RGC (dRGCs) are located in the inner nuclear layer (INL). A well-known example RGC group which frequently exhibits displaced cell bodies are intrinsically photosensitive ganglion cells (ipRGCs) in various mammalian retinas including human^[e.g.17–24^. ipRGCs express the photopigment melanopsin and mediate distinct functions in image- and non-image forming vision^25^. In the mouse retina, six different types of ipRGCs (M1-M6) can be separated based on morphology, functional properties, and axonal projections to distinct brain regions^18,23,26–31^. The M4 ipRGC was shown to correspond to one of the four alpha RGC types of the mouse retina, i.e. the sustained ON alpha (sONɑ) RGC^29,32^.

Despite the knowledge of this heterogeneous group of ipRGCs and the functional roles of corresponding types, little is known about the properties of displaced ipRGCs. In mice, about 2% of all RGCs are located as displaced retinal ganglion cells (dRGCs) in the inner nuclear layer (INL). They occur more frequently in the retinal periphery^12^. Individual dRGCs show a large morphological variety^33^ but dRGCs have not been comprehensively examined with regard to cell identity and regularly placed RGCs. Thus, the spatial arrangement of their dendritic processes with respect to regular RGCs in the GCL or their response properties remain unknown. While dRGCs are often ignored when RGC mosaics are investigated in the mammalian retina, it is thought that dRGCs are misplaced due to ontogenetic aberrations rather than representing an independent functional class of RGCs^3,34^. In birds, however, dRGCs mostly project to the accessory optic system and presumably support the optokinetic nystagmus and retinal image stabilization^35–37^.

To elucidate the anatomical and functional arrangement of dRGCs relative to mosaics of regular RGCs in a mammalian retina, we took advantage of available immunomarkers against the group of ipRGCs in mice. We analyzed the spatial distribution and dendritic arrangement of the ipRGC types M1, M2, and sONɑ, in both GCL and INL of the retina. dRGCs completed the dendritic mosaic and tiled the retina evenly with their regularly placed partners to form complete anatomical and functional mosaics. In addition, we found that the distribution of identified dRGCs neither followed the overall density distribution of all RGCs, nor the distribution of the corresponding type in the GCL. Furthermore, the proportion of displaced cells differed between types. Our data suggest that a displacement of cell bodies does not occur randomly but in a type-dependent manner.

## Materials and methods

### Animals and tissue preparation

All procedures were performed in accordance with the German Animal Protection Law (*Tierschutzgesetz*), as issued by the German Federal Government, approved by the local animal welfare officers, in compliance with the AVMA Guidelines for Euthanasia, and reporting adheres with the ARRIVE guidelines. Wild type C57Bl6/J mice and *thy1-*GFP-O mice^38^ of either sex were included in this study. They were housed in a 12:12 h light dark cycle. Fully grown C57Bl6/J animals at an age of 11–12 weeks were used for cell quantifications. The animals were euthanized using carbon dioxide followed by a decapitation. Eyes were enucleated and fixed in 4% cold paraformaldehyde in 0.01 M phosphate buffered saline (PBS), pH 7.4, for 15 min at room temperature. After washing in PBS, special care was taken to maintain the orientation of the eyecups, where the choroid fissure was used as a landmark^39^. Four radial relieving cuts were made, the longest one along the nasal fissure, approaching the optic nerve head. Then, the retinas were dissected away from the eye cup in PBS. This was performed in a way that the complete retina, including the most peripheral region (outer marginal zone), was preserved. Retinas were then cryoprotected overnight with 30% sucrose in PBS, and stored at -20 °C until use.

*thy1-*GFP-O mice at an age of 5–7 weeks were used for multi-electrode array (MEA) recordings. Animals were dark adapted for 2 h and killed by cervical dislocation. Dorsal-temporal regions of the retinas were dissected from the eye cup under infrared illumination in Ames’ solution, pH 7.4 (USBiological) bubbled with carbogen (95% O_2_ and 5% CO_2_) at room temperature. MEA recordings were performed at 36.5 °C in the recording chamber.

### Immunohistochemistry

Following dissection, retinas were used as whole mounts. Immunohistochemical labeling was performed by an indirect immunofluorescence method. The tissue was mounted on a black nitrocellulose filter membrane (Millipore) with GCL up and pre-incubated at room temperature for 2–4 h in an incubation solution containing 5% NDS, 1% BSA, 1% Triton X-100, and 0.02% sodium azide dissolved in PBS. Primary and secondary antibodies were diluted in the same incubation solution. Wholemounts were incubated with primary antibodies (Table 1) for 3 days at room temperature. Secondary antibodies (Alexa 488 and Alexa 568, Invitrogen, 1:500; Alexa 647, Invitrogen or Jackson ImmunoResearch, 1:250) were incubated at room temperature for 4 h. 4′,6-Diamidino-2-phenylindole dihydrochloride (DAPI, Sigma-Aldrich, cat# D9542, final concentration 0.2 µg/ml) was added into the secondary antibody solution for 1 h before rinsing.

The tissue was mounted on glass slides and coverslipped with Vectashield (Vector Laboratories). Spacers between glass slides and coverslips were used to avoid squeezing the tissue. High-resolution image stacks were taken with confocal laser scanning microscopes (Leica TC SP8 and Leica TCS SL) with 40x/1.25 oil immersion objectives and z-axis increments of 0.25-0.5 μm or with a Zeiss AxioObserver equipped with an Apotome2 and a 20x/0.8 air objective and z-axis increments of 0.54 μm.

**Table 1:** List of primary antibodies.

Primary antibody	Host	Company	Catalogue number	Dilution
RBPMS	Rabbit	PhosphoSolutions	1830-RBPMS	1:500
RBPMS	Guinea pig	PhosphoSolutions	1832-RBPMS	1:200
SMI-32 (Neurofilament H)	Mouse	BioLegend	801701	1:1000
Calbindin	Rabbit	Swant	CB-38a	1:2000
Osteopontin	Goat	R&D systems	AF1433	1:200
Melanopsin	Rabbit	ATS	AB-N38	1:2500
Melanopsin	Rabbit	ATS	AB-N39	1:2500
GFP	Rabbit	Invitrogen	A-6455	1:2000

Cell bodies labeled with RBPMS, SMI-32 and calbindin or melanopsin were manually outlined and the corresponding area was measured in Fiji to calculate their diameter.

For the calculation of density recovery profiles^40^ and for large-scale quantification of cell densities, tile scans of image stacks from whole retinas were taken with a Leica DM6 B epifluorescence microscope equipped with a motorized stage and a 20x/0.5 air objective. Individual image stacks were automatically stitched together in the microscope software (LAS X, Leica). Next, the centers of individual cell bodies were manually marked using the *CellCounter* plugin in Fiji.

### Reconstruction of retinal wholemounts

A number of landmarks that were used in the spherical reconstruction and density calculations were marked in Fiji and Matlab. These include the outline of the flattened retina along with additional marks for cuts and tears within the said outline, the optic disc, areas where the retina ruptured internally, areas where tissue was missing, as well as prominent anatomical features such as blood vessels. The outline was estimated in rare cases where tissue was folded or missing. The R package Retistruct^41^ was then used to reconstruct the dissected retina into the partial sphere formed by the retina in the eye. The complete retina was estimated to form a spherical cap with a rim angle of 110°^41^. The vascularisation pattern was used to assess the quality of the reconstruction. Orientations of different retinas were aligned based on the incision along the nasal choroid fissure. The average nasal choroid fissure incision line and the average optic disc is shown in Fig. 7C. The center of the optic disc was ventrally offset from the pole of the cap with an angle of 5.2 ± 2.6°. The radius of the retinal cap, in particular of the ganglion cell layer, was set to 1.5 mm as measured in cryosections of complete mouse eyes. This is in accordance with previous reports^42^. We discarded the radius estimated by equalizing the area of the cloverleaf and spherical cap (1.3 ± 0.0 mm, *n* = 10). The radius difference was likely caused by an underestimation of the area of the flattened whole-mounted retina due to potentially compressed tissue in the center of the cloverleaf around the optic nerve head while unfolding the retina during the mounting procedure.

The local cell density (i.e. density of cell body markers) was calculated in spherical coordinates. The density at a given position was the number of markers in the local neighborhood within a 10 degree radius. This radius corresponds to an arc length of 261.8 μm and a counting disc area of 0.21 mm^2^. Regions such as the optic disc or regions with insufficiently stained or damaged tissue were excluded from contributing to the area. The average density across different retinas (Fig. 7) was calculated at a fixed regular grid. Reconstructed points were visualized as azimuthal equal distance projection (Fig. 7).

### Matching of anatomically and electrophysiologically identified cells

Retinas were recorded as described previously^43^. Briefly, a small piece of isolated retina from the dorso-temporal quadrant of the eye was mounted, RGC side down, on a high-density MEA (3Brain). Recordings were analyzed offline to isolate the spikes of different cells. Candidate spike events were detected using a threshold on each electrode. The voltage waveforms on the electrode and neighboring electrodes around the time of the spike were extracted. Clusters of similar spike waveforms were identified as candidate neurons if they exhibited a refractory period. Duplicate recordings of the same cell were identified by temporal cross-correlation and removed. A monochrome binary spatial white noise was displayed on a CRT monitor at a refresh rate of 120 Hz and a stimulus pixel width of 49 μm. The grid on which stimulus pixels were presented was randomly shifted, effectively doubling the spatial resolution. Photopic light levels at a mean intensity of 2.9 mW/m^2^ were used to characterize the response properties of the recorded cells. The receptive field was approximated by the spike-triggered average. RGCs were functionally classified into cell types based on their spatiotemporal receptive field properties and spike autocorrelation function given that they formed a regular mosaic. sONɑ RGCs were identified based on their sustained responses, large receptive fields, and response pattern to full field frequency- and amplitude-modulated sweeps^1^. Receptive field outlines were drawn at the 1 s.d. contour of two-dimensional Gaussian fits. The electrical image was calculated as the average voltage recorded in a 5.1 ms time window surrounding the spikes of a cell^44^.

After the MEA recordings, a manual tile scan was performed while the retina was still mounted on the MEA. Images were obtained with a Leica DMLFS epifluorescence microscope equipped with a 10x air objective and stitched together in Fiji using the pairwise stitching plugin. Subsequently, the tissue was carefully mounted on a black nitrocellulose filter membrane (Millipore) with the RGC layer up and processed for immunostainings as described above. Mosaics of sONɑ RGCs were identified independently by immunolabeling and functional analyses as described above. GFP-labeled sONɑ RGCs were traced with Amira (Thermo Fisher Scientific) in the epifluorescence image from the retina when it was still mounted on the MEA to match GFP-labeled cells and the readily visible electrodes together with the corresponding electrical image. A unique mapping of anatomical and electrophysiological data was achieved by the comparison of the cell body and axon positions with the corresponding voltage deflections in the electrical images among all recorded cells.

Unless stated otherwise, all data are reported as mean ± s.d. (standard deviation).

## Results

**Fig. 1:**
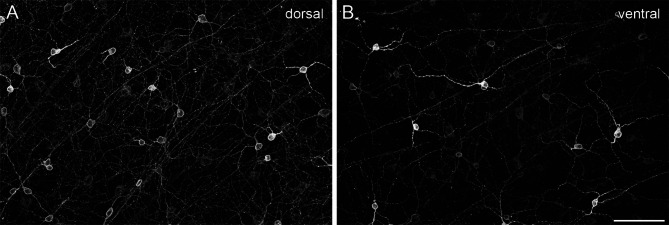
Non-uniform staining pattern of melanopsin across the mouse retina. Maximum intensity projections of structured illumination epifluorescence images labeled with melanopsin were taken from a dorsal (**A**) and a ventral (**B**) region of the same retina. Scale bar: 50 μm.

Mouse retinas were labeled against melanopsin in combination with further immunomarkers to investigate the distribution of regularly placed ipRGCs and displaced cells. The melanopsin immunolabeling revealed a non-uniform staining pattern across the mouse retina as shown previously^25,45^. In the dorsal retina, cell bodies and dendrites of M1 and M2 ipRGCs were clearly visible with a rather homogeneous melanopsin staining level. In contrast, the melanopsin staining pattern in the ventral retina was less homogeneous, generally weaker in most cells, but with strongly labeled (M1) cells that stood out (Fig. 1). Thus, further markers were required to reliably identify cell types and their specific distribution patterns across all retinal regions.

### Osteopontin labels M2 ipRGCs

**Fig. 2:**
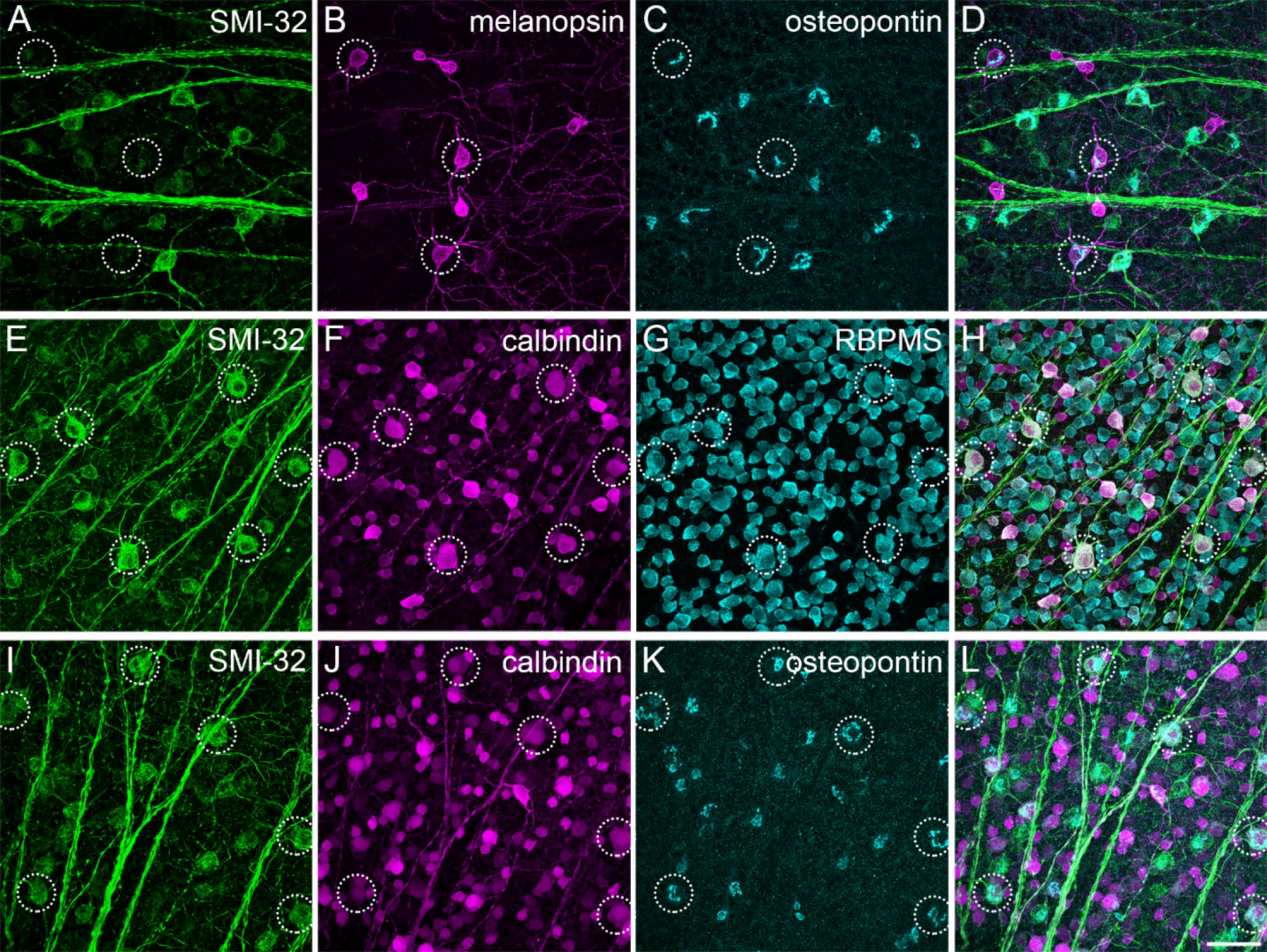
Immunomarkers for ipRGC/sONɑ cell type classification. Maximum intensity projections of confocal image stacks of the mouse GCL triple-labeled against the neurofilament marker SMI-32, melanopsin, calcium-binding protein calbindin, RNA binding protein with multiple splicing (RBPMS), and osteopontin in different combinations. (**A**)–(**D**): Cell bodies of several melanopsin-positive ipRGCs are immunonegative for SMI-32 but labeled with osteopontin (dashed circles). (**E**)–(**H**): Cell bodies of sONɑ RGCs (dashed circles) are co-labeled with SMI-32, calbindin, and RBPMS. (**I**)–(**L**): As (**E**)–(**H**) but with osteopontin instead of RBPMS. Scale bar: 50 μm.

**Fig. 3:**
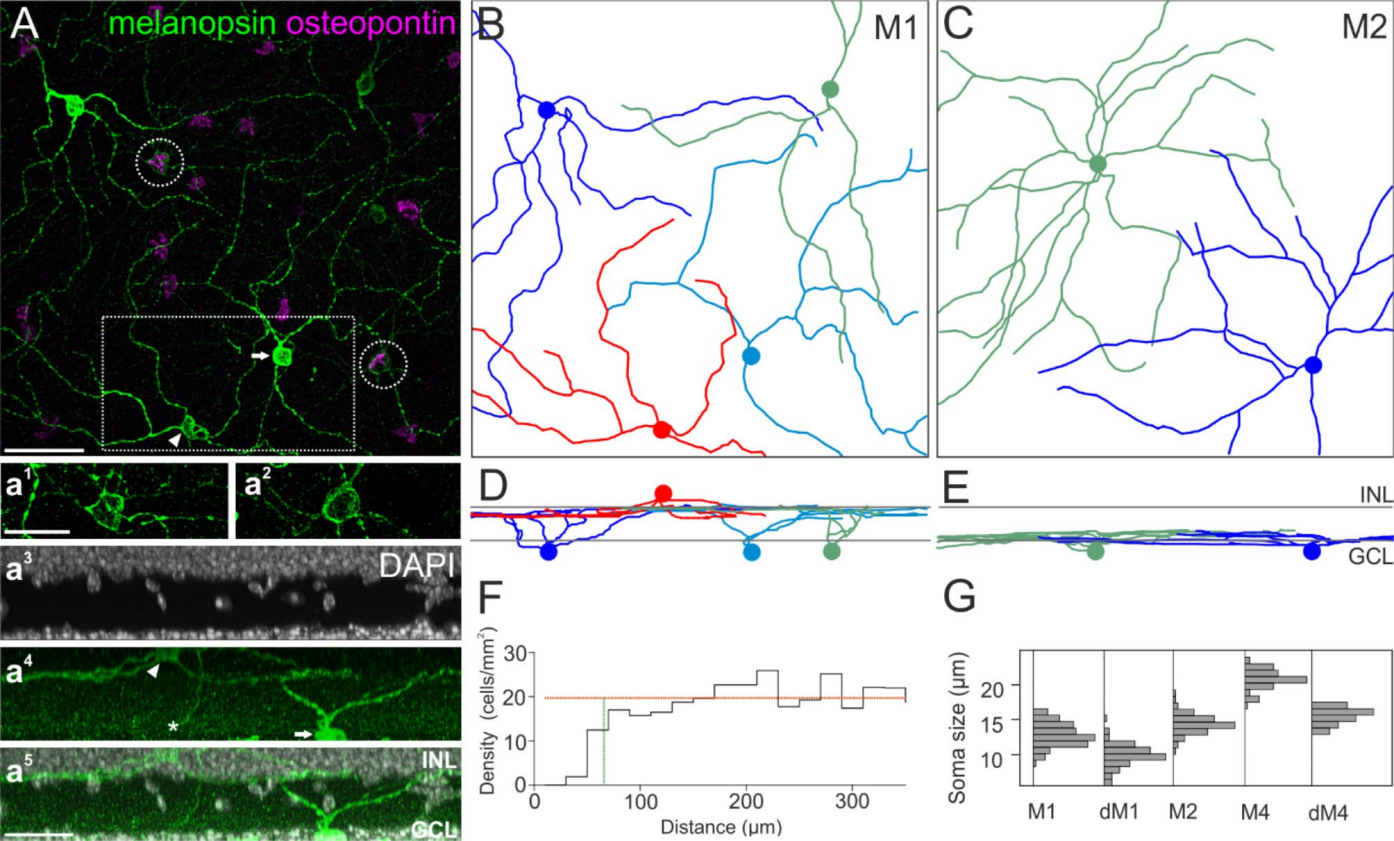
Tiling pattern and stratification levels of osteopontin-negative (M1) and -positive (M2) ipRGCs. (**A**) Maximum intensity projection of a confocal image stack of wholemounted retina labeled against melanopsin and osteopontin. Dendrites of M1 and M2 ipRGCs were manually traced through the image stack in Amira. a^1^ and a^2^ show the staining pattern of melanopsin labeled cell bodies from A (dashed circles) with increased intensity. a^3^–a^5^: Side view projection of the boxed area in A including the DAPI staining, indicative of the inner nuclear layer (INL) and ganglion cell layer (GCL). The arrowheads (**A**, a^4^) point to the cell body of a displaced M1 cell in the INL, the asterisk (a^4^) marks the descending axon. The arrows (**A**, a^4^) point to an M1 cell with its cell body located in the GCL. (**B**) Ganglion cell skeletons of M1 ipRGCs. Colored discs indicate the positions of the cell bodies. The red skeleton at the bottom of (**B**) belongs to a displaced ganglion cell. (**C**) Ganglion cell skeletons of M2 ipRGCs. (**D**), (**E**) vertical views of (**B**), (**C**). Gray lines indicate the inner and outer border of the IPL as determined by DAPI staining. (**F**) Density recovery profile of M2 cells (*n* = 281) analyzed in a wholemounted retina shows the distribution of distances between M2 ipRGCs. Effective radius of exclusion (vertical green line): 65.8 μm, cell density average over the larger half of the bins (horizontal orange line): 19.71 cells/mm^2^, bin width 20 μm. (**G**) Histogram of ipRGC body diameters, M1 *n* = 114; dM1 *n* = 81; M2 *n* = 81; M4/sONɑ *n* = 75; displaced M4/sONɑ *n* = 21 (all peaks normalized to equal amplitude). Scale bars: 50 μm in A, applies to (**A**)–(**E**); 25 μm in a^1^ and in a^5^.

M1 and M2 ipRGCs are strongly immunopositive for melanopsin but a specific marker to clearly separate these two types is missing. The cytosolic protein osteopontin^46^ is commonly used as an alpha RGC marker in mice, but it has been suggested that another type of melanopsin-containing RGCs is immunoreactive against osteopontin as well^2,47^. Thus, we analyzed the staining pattern of osteopontin in combination with melanopsin and further cell marker antibodies to determine if these combinations were suitable for a reliable separation of the RGC types of interest (Fig. 2A-D, encircled). We used SMI-32^11,48^ and calbindin^47^ to rule out a confusion with sONɑ RGCs (Fig. 2E-L, encircled), that express osteopontin and low levels of melanopsin^29,32^ (and own observations, Fig. 2). In addition, RBPMS (RNA-binding protein with multiple splicing^49^), a general ganglion cell body immunomarker, was applied to further elucidate general anatomical properties of RGC bodies. sONɑ RGCs had extraordinarily large and polygonal-shaped cell bodies (Fig. 2F, G, soma diameter 20.7 ± 1.3 μm; *n* = 75; see also Fig. 3G).

The marker combination presented here allowed the unequivocal classification of sONɑ RGCs as the latter were very large, strongly positive for SMI-32, and only weakly positive for melanopsin. Thus, at least one other RGC type was also immunoreactive for both osteopontin and melanopsin. To identify this type, we used dendritic stratification to classify RGCs co-labeled with both osteopontin and high levels of melanopsin. The dendrites of M1 or M2 ipRGCs stratify in sublamina S1 or S5 of the inner plexiform layer (IPL), respectively^50^. If the immunoreactivity against osteopontin in melanopsin-labeled ipRGCs was a type-specific feature, this should be revealed by distinct stratification patterns of the corresponding cells. Thus, we analyzed the dendritic stratification of osteopontin-positive and -negative ipRGCs, which were strongly melanopsin-positive. These cells were manually traced through microscopic image stacks of immunolabeled wholemounts (Fig. 3). Cell body positions and dendritic stratification levels were determined relative to DAPI staining as an indicator for the ganglion cell layer and the inner nuclear layer here (Fig. 3a^3^) and in subsequent experiments.

Cells that were negative for osteopontin but strongly positive for melanopsin exclusively stratified in the most distal part of the IPL (sublamina S1, Fig. 3D). These cells had sparsely branched dendritic arbors and relatively small cell bodies (Fig. 3G, soma diameter 12.8 ± 1.6 μm; *n* = 114). We identified these cells as M1 ipRGCs based on staining intensity, overall morphology, and stratification level. In contrast, osteopontin-positive, strongly melanopsin labeled ipRGCs exclusively stratified in the most proximal part of the IPL (sublamina S5, Fig. 3E). Cell bodies of these cells were slightly larger compared to M1 ipRGCs (soma diameter 14.4 ± 1.3 μm; *n* = 81). They had symmetric dendritic trees and more branchpoints than M1 ipRGCs, similar to the morphology that was described for M2 ipRGCs^20^.

All M2 candidate cell body positions (*n* = 281) were manually marked in an immunolabeled retina and a density recovery profile was calculated. An exclusion zone with an effective radius of 65.8 μm became apparent (Fig. 3F). This exclusion zone further supported the notion that these cells formed a single type which tiled the retina (see also Fig. 7M-O).

Together, these results showed that strongly melanopsin labeled cells, which were osteopontin-positive and SMI32-negative, represented M2 ipRGCs.

### Dendritic tiling pattern of regular and displaced RGC types

**Fig. 4:**
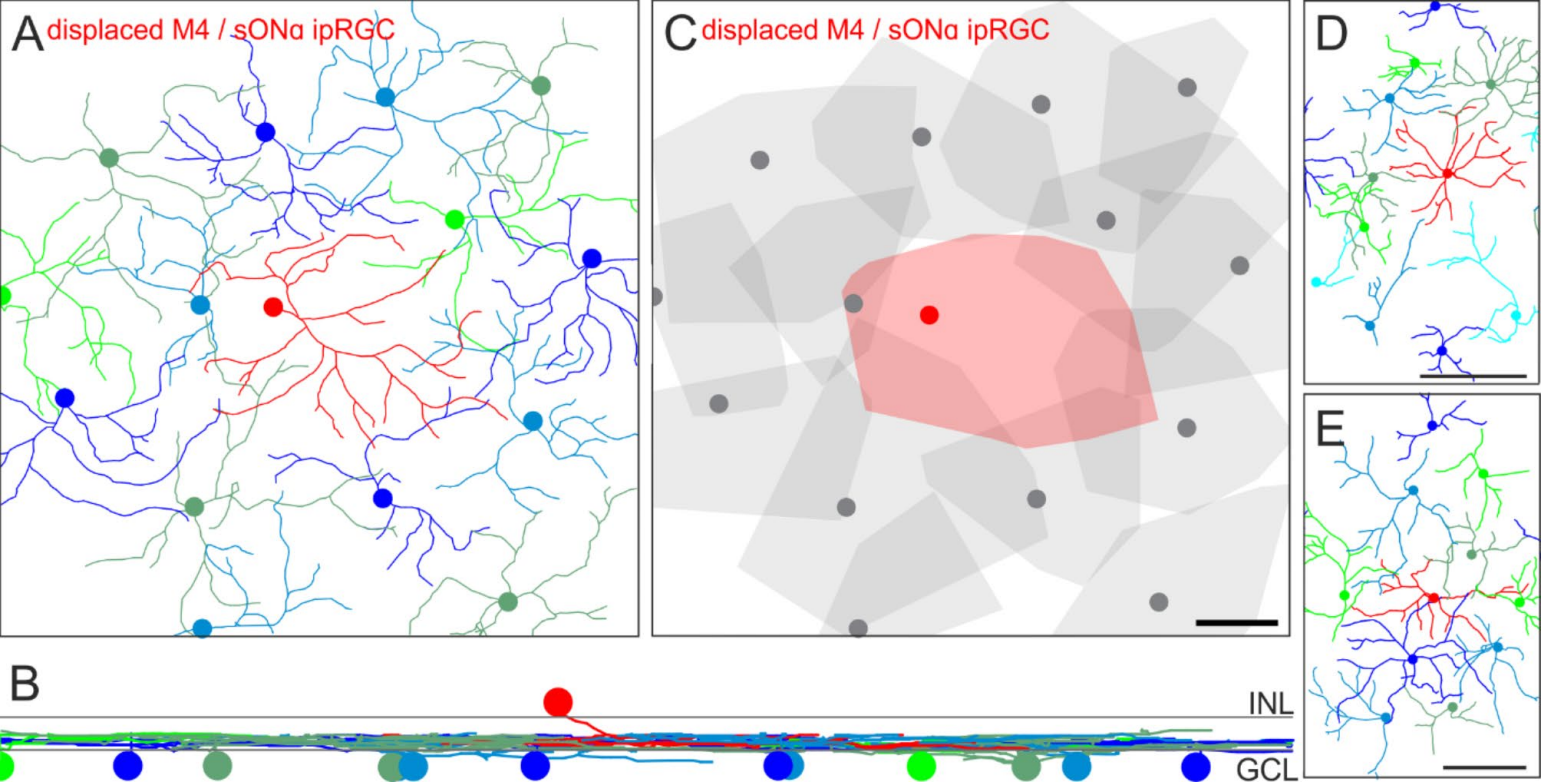
Tiling pattern and stratification levels of M4/sONɑ RGCs. Dendrites of sONɑ RGCs were manually traced through a confocal image stack of a mouse retina labeled against SMI-32 and DAPI. (**A**) sONɑ RGC skeletons. Colored discs indicate the positions of the cell bodies. The red skeleton in the center belongs to a displaced sONɑ RGC. (**B**) Vertical view of the RGC skeletons in A. Gray lines indicate the inner and outer border of the inner plexiform layer as determined by DAPI staining. INL: inner nuclear layer, GCL: ganglion cell layer. (**C**) Convex hulls encompass the skeletons as in (**A**). The displaced cell is shown in red. Note that dendritic trees of some ganglion cells in the periphery of the image stack were not fully captured. Scale bar in (**C**): 50 μm, also applies to (**A**). (**D**), (**E**) as (**A**) for additional examples from two further animals. Scale bars 500 μm.

We analyzed the spatial arrangement of dendritic processes of displaced RGCs and their regular counterparts in the GCL to understand the dendritic organization relative to each other. For this, we used SMI-32 staining in confocal image stacks as in Fig. 2. SMI-32 staining is very dense in the IPL as it labels processes of many different cell types. Therefore, we manually traced the dendritic trees of displaced RGC types and those of their regularly placed neighbors of the same type through the image stacks (Fig. S1, Fig. 4).

The red dendritic skeleton originates from a displaced sONɑ RGC (Fig. 4A, B). The cell bodies of all remaining cells were located in the GCL. Even though SMI-32 does not reliably label the most peripheral dendrites of a given tree, it became obvious that all of these cells stratified at the same level in the ON layer of the IPL. Furthermore, displaced sONɑ RGCs completed a hole in the mosaic of their regularly placed partners, and together they evenly tiled the retina (Fig. 4C).

**Fig. 5:**
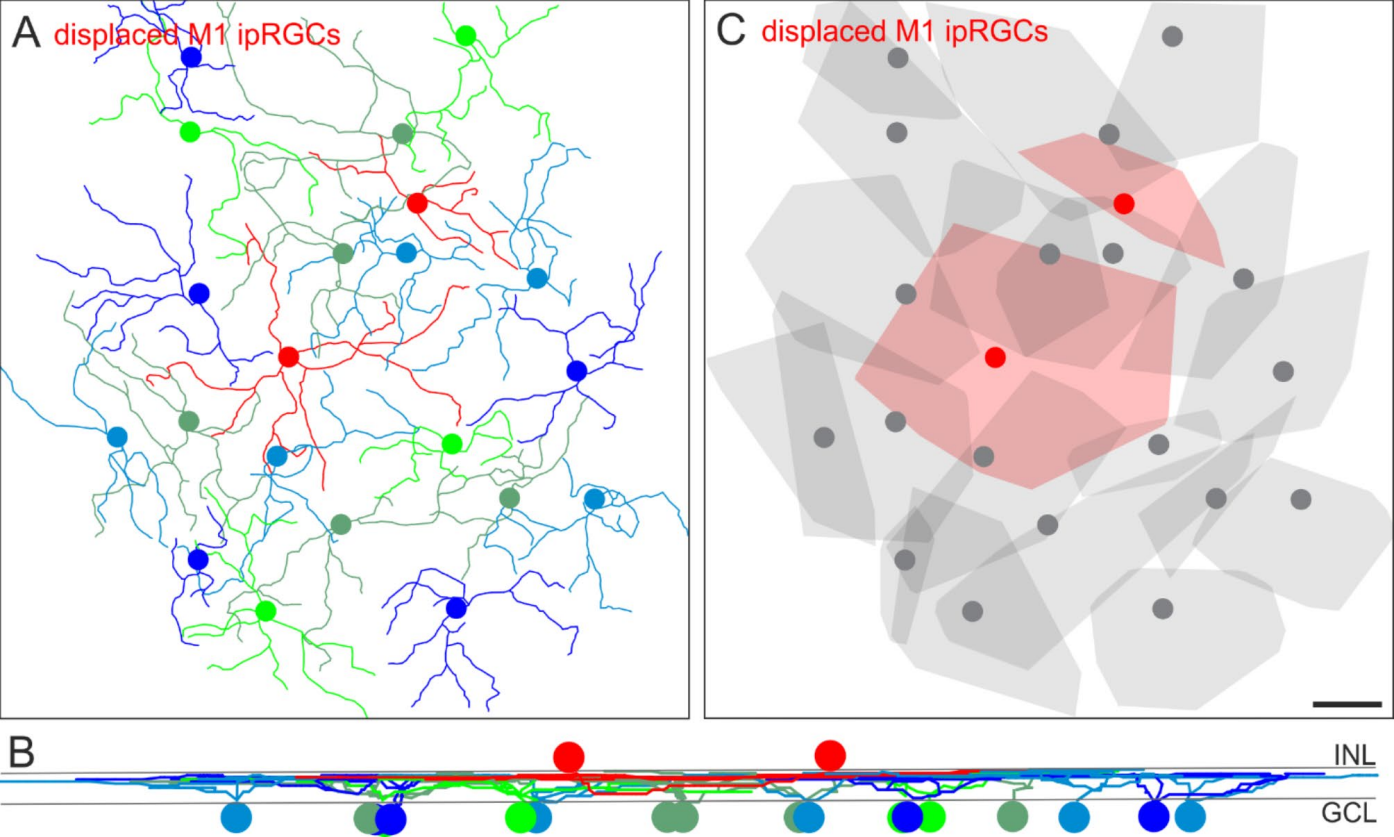
Tiling pattern and stratification levels of M1 ipRGCs. (**A**) Melanopsin immunostaining was used to trace the dendrites of M1 cells through the image stack in Amira. The resulting M1 ipRGC skeletons are shown. The red skeletons represent tracings of displaced M1 cells. (**B**) Vertical view of the ganglion cell skeletons in (**A**). INL: inner nuclear layer, GCL: ganglion cell layer. (**C**) Convex hulls encompass the skeletons as in (**A**). Displaced cells are shown in red. Note that dendritic trees of some ganglion cells in the periphery of the image stack were not fully captured. Scale bar in C: 100 μm, also applies to (**A**).

To ensure that this dendritic tiling pattern of displaced cells is not exclusively related to sONɑ RGCs, we also analyzed the putative integration of displaced M1 ipRGC dendrites within the mosaic of regularly placed M1 ipRGCs in a ventral mouse retina (Fig. 5). The red skeletons in the center of Fig. 5A indicate displaced M1 ipRGCs with cell bodies located in the INL. These cells completed the mosaic of the remaining, regular counterparts with their cell bodies located in the ganglion cell layer. All M1 ipRGCs stratified at the same level in the distal OFF layer of the IPL (Fig. 5B). As shown for sONɑ RGCs, displaced M1 ipRGCs completed the mosaic of their regularly placed partners of the same type (Fig. 5C).

### Receptive field mosaic of regular and displaced sONɑ RGCs

We presented anatomical evidence that displaced RGCs tile the retina in concert with their regularly placed counterparts of the same type to form complete dendritic mosaics. This spatial pattern should be reflected in a homogeneous, uninterrupted mosaic of the functional receptive fields when a displaced ganglion cell is present, assuming that displaced RGCs would not differ from regular cells in terms of their light response properties.

**Fig. 6:**
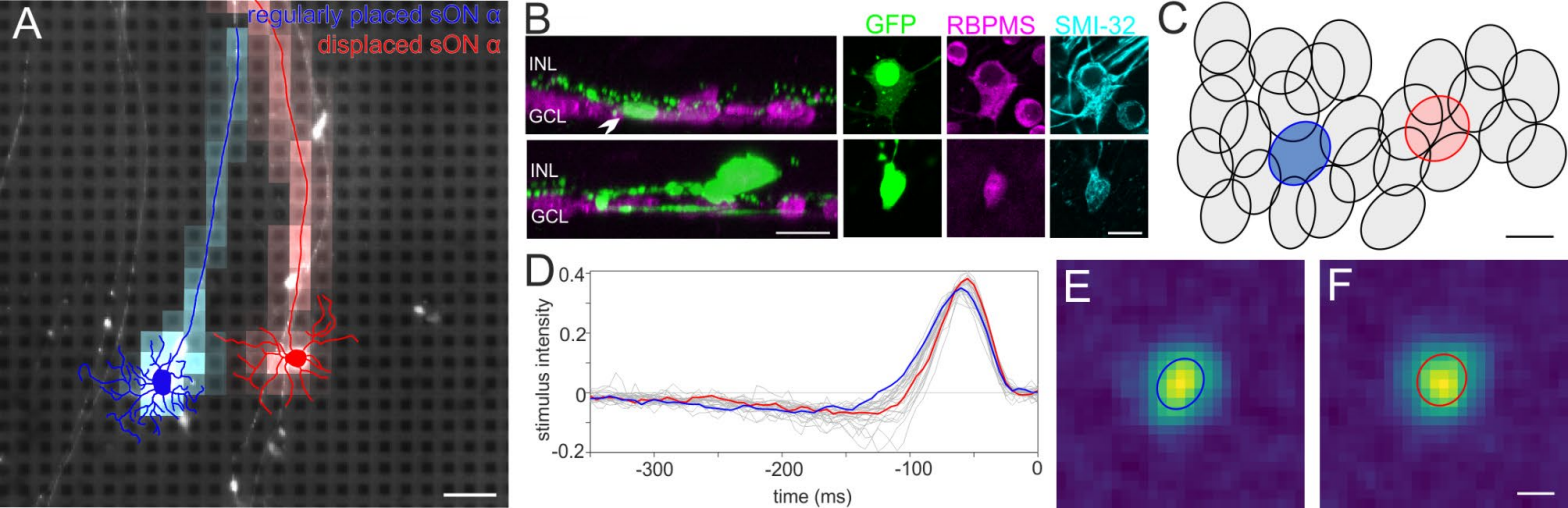
Displaced and regular sONɑ RGCs share similar light response properties. (**A**) Epifluorescence image of the recorded GFP-O retina on the MEA shows native GFP fluorescence in a regularly placed sONɑ (traced in blue) and a displaced sONɑ RGC (traced in red), overlayed with the respective electrical image. Scale bar 100 μm. (**B**) Projections of confocal image stacks of the same cells from A after staining with antibodies against RBPMS, GFP, and SMI-32. Left panels: Side view projections of cell bodies (arrowhead) and dendrites of regularly placed sONɑ (top) and displaced sONɑ RGC (bottom). INL: inner nuclear layer, GCL: ganglion cell layer. Right panels: Top (wholemount) view projection of GFP, RBPMS and SMI-32 staining of cell bodies from the marked cells in the left panels (and from (**A**)). Both cells are strongly immunopositive for SMI-32. Scale bars 20 μm. (**C**) Receptive field outlines of the sONɑ RGC mosaic. Blue and red ellipses indicate the receptive field fits of the regularly placed and displaced sONɑ RGCs in (**A**). Scale bar 100 μm. (**D**) Spike-triggered average stimulus time courses of all sONɑ RGCs shown in (**D**). Blue and red lines indicate the time courses of the regularly placed and displaced RGC from (**A**), respectively. (**E**) Spatial receptive field of the regularly placed sONɑ RGC shown in (**A**)-(**D**). (**F**) Spatial receptive field of the displaced sONɑ RGC shown in (**A**)-(**D**). Scale bar 100 μm.

**Fig. 7:**
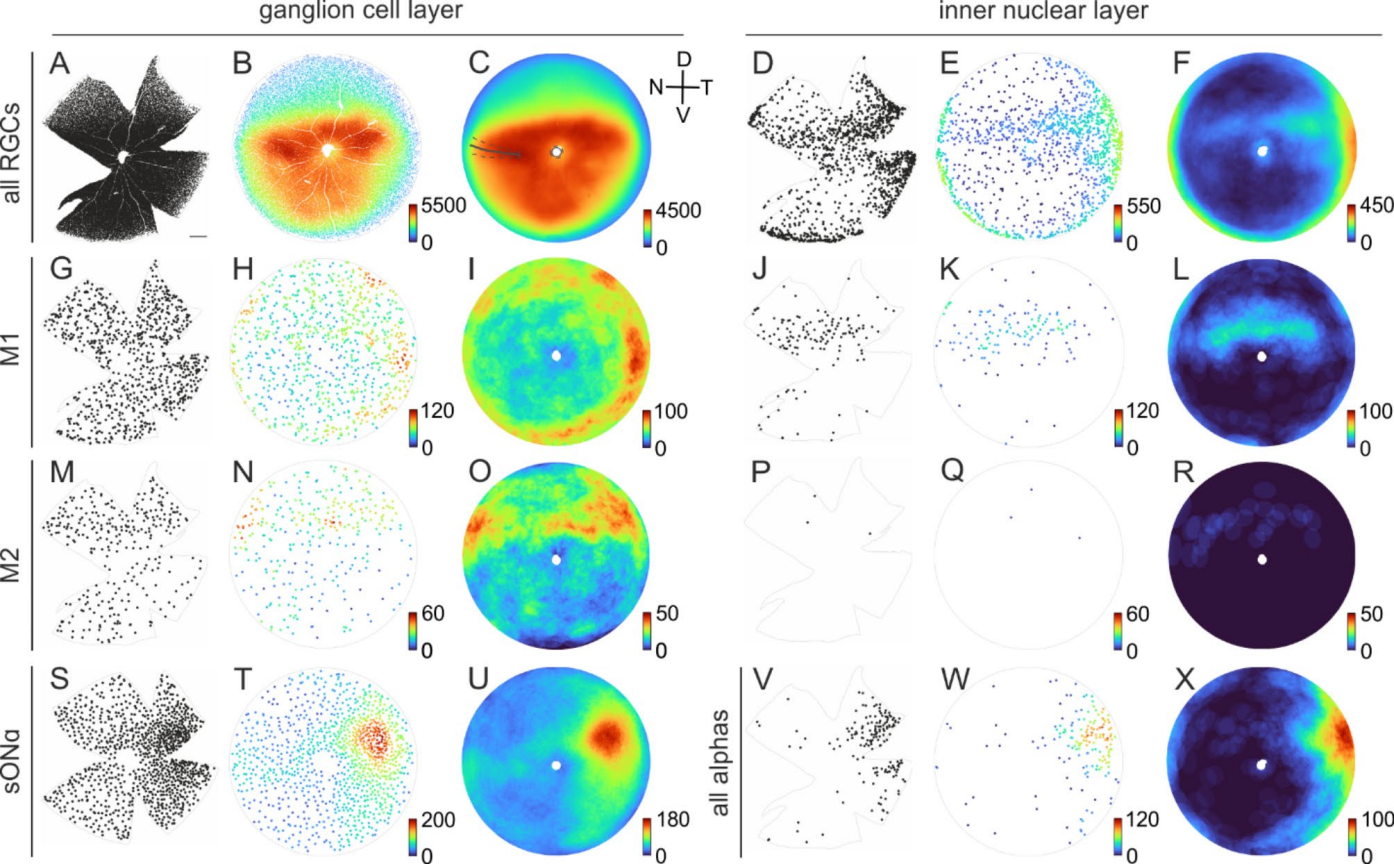
Population densities across the retina. (**A**) Each dot indicates the position of an individual RGC with its cell body located in the ganglion cell layer of a retina (gray outlines) from a left mouse eye, labeled with the RGC marker RBPMS. Scale bar in (**A**) 500 μm, applies to all cloverleaf-shaped samples. (**B**) Azimuthal equal-distance projection of the reconstructed retinal sphere. The color of the dots represents the local density within a radius of 10 deg (spherical distance of ~ 262 μm) of the corresponding cell. Color bar: density of cells/mm^2^. (**C**) Density distributions as in (**B**), averaged across 4 retinas. For the averages across multiple retinas, the retina orientation was adjusted based on the individual incisions along the nasal choroid fissure. Solid gray line: average fissure incision for all retinas used for analysis (*n* = 10); dashed line: standard deviation. (D = dorsal, V = ventral, N = nasal, T = temporal) (**D**)–(**F**) as (**A**)–(**C**) for displaced RGCs. (**G**)–(**L**) as (**A**)–(**F**) for M1 ipRGCs (melanopsin positive, osteopontin negative), averaged across 5 retinas. (**M**)–(**R**) as (**A**)–(**F**) for M2 ipRGCs (melanopsin positive, osteopontin positive), averaged across 5 retinas. (**S**)–(**U**) as (**A**)–(**C**) for sONɑ RGCs in the GCL (SMI-32 positive, calbindin positive, osteopontin positive) and (**V**)–(**X**) as (**D**)–(**F**) for all ɑRGCs in the INL (melanopsin negative, osteopontin positive), averaged across 4 retinas. All alpha RGCs instead of sONɑ were analyzed for technical reasons, see Results for further explanations.

To test this hypothesis, the receptive field organization of sONɑ RGCs was characterized by MEA recordings of spikes from a GFP-O mouse retina in response to a random noise light stimulus (Fig. 6). GFP-O retinas were used to identify GFP-positive sONɑ RGCs^51^. We recorded from the dorso-temporal retina to increase chances of recording from a displaced sONɑ RGC (see Fig. 7V-X) among regular RGCs. Two nearby GFP-positive RGCs were recorded simultaneously (Fig. 6A), which were subsequently confirmed as a regularly placed sONɑ (traced in blue) and a displaced sONɑ RGC (traced in red), based on dendritic tree morphology, strong SMI-32 labeling, and the locations of cell bodies in GCL and INL, respectively (Fig. 6A, B). These two anatomically identified cells were then matched to the functional dataset from the MEA recording, by screening all candidate electrical images of all RGCs recorded in the corresponding area. We found matching pairs for both cells (Fig. 6A) based on soma positions and axon trajectories. The cells were part of the sONɑ functional mosaic (Fig. 6C) which was identified independently (see material and methods). The regularly placed and displaced sONɑ RGCs show similar receptive field properties in terms of spike response kinetics (Fig. 6D-F). The receptive field radius of the displaced RGC did not deviate significantly from those of regularly placed RGCs (Wilcoxon ranksum *p* > 0.74). In line with our anatomical results, the receptive field of the displaced sONɑ RGC (red ellipse in Fig. 6C) completed the receptive field mosaic of its presumably regular-placed counterparts (gray ellipses).

### Distribution of RGC types across the retina

Here, dRGCs were observed more frequently than expected in areas other than the outer marginal zone^12^. Therefore, we investigated the general distribution patterns of dRGCs relative to regular RGCs across complete retinas and in a type-specific manner by a large-scale analysis of the abovementioned immunomarkers (Table 2).

We applied these marker combinations for a localization analysis of RGC bodies in immunolabeled wholemounts (Fig. 7). Stress-relief cuts to flatten the tissue yielded the typical cloverleaf-like shape of the samples. One of these cuts was placed exactly along the nasal choroid fissure during dissection for orientation of the retina. We manually counted each cell body in a given marker combination across complete retinas (see Methods), and their positions are indicated by dots in Fig. 7 (black dots in left columns of GCL and INL panels). We reconstructed the cloverleaf shape of the tissue back into the original, almost hemispherical structure of the eyecup with Retistruct to calculate the densities of cells across the complete retinas^41^.

**Table 2:** Cell counts.

Animal #	RGCs in the GCL	dRGCs in the INL	Total number of RGCs
1	49,199	1200	50,399
2	50,226	1273	51,499
3	51,990	1318	53,308
4	56,369	1265	57,634
Mean	51,946	1264	53,210
SD	3166	49	3184

	M1 ipRGCs in the GCL	Displaced M1 ipRGCs in the INL	Total number of M1 ipRGCs
1	847	156	1003
2	800	173	973
4	833	139	972
5	708	138	846
6	837	164	1001
Mean	805	154	959
SD	57	15	65

	M2 ipRGCs in the GCL	Displaced M2 ipRGCs in the INL	Total number of M2 ipRGCs
1	356	11	367
2	384	2	386
4	302	3	305
5	281	2	283
6	356	9	365
Mean	336	5	341
SD	43	4	45

	sONɑ RGCs in the GCL		
7	1029		
8	1092		
9	1070		
10	981		
Mean	1043		
SD	49		

	ɑRGCs in the GCL	Displaced ɑRGCs in the INL	Total number of ɑRGCs
1	2285	193	2478
2	2367	218	2585
3		247	
4	2449	164	2613
Mean	2367	206	2559
SD	82	35	71

The local density in 3D was projected into an azimuthal equal distance plot and is indicated by the color of the dots (Fig. 7, colored dots in middle columns of GCL and INL panels). Density calculation in the reconstructed retinas allowed us to observe the averaged data of 4 or 5 retinas per analysis type (Fig. 7, colored discs in the right columns of GCL and INL panels).

### Distribution of RGC types across the retina—GCL

We identified 51,946 ± 3165 RGC bodies throughout the GCL of complete retinas (*n* = 4), which was largely consistent with previous reports^12,13^. RGCs were not uniformly distributed throughout the mouse retina (Fig. 7A-C). Beyond a general central-to-peripheral gradient, the density distribution showed a steep increase in the dorsal half toward an area of highest RGC density above the optic disc and in the ventral retina.

The distribution of M1, M2, and sONɑ RGCs in the ganglion cell layer did not follow the overall density gradient of all RGCs and showed type-specific distribution patterns. 805 ± 57 M1 ipRGCs per retina (*n* = 5) were counted (Fig. 7G-I). This represented a fraction of 1.6 ± 0.2% of all RGCs in the GCL. They had a higher density in the dorsal and temporal periphery. M2 ipRGCs showed areas of higher density in the dorsal retina (*n* = 5, Fig. 7M-O)^45^. We counted 336 ± 43 M2 ipRGCs per retina. These were 0.7 ± 0.1% of all RGCs in the GCL. sONɑ RGCs had a density peak in the dorso-temporal retina^11^ (Fig. 7S-U). We counted 1043 ± 49 sONɑ RGCs per retina (*n* = 4). These were 2.0 ± 0.2% of all RGCs in the GCL.

### Distribution of ipRGC types across the retina—INL

The cell bodies of dRGCs were located in the most proximal part of the INL. They were distinguished from amacrine cells by their larger cell bodies and RBPMS immunoreactivity. However, the cell body size of dRGCs was significantly smaller compared to that of their regularly placed counterparts (Fig. 3G; soma diameters: M1 12.8 ± 1.6 μm, *n* = 114; displaced M1 10.0 ± 1.6 μm, *n* = 81 (Wilcoxon ranksum *p* < 0.001); sONɑ 20.7 ± 1.3 μm, *n* = 75; displaced sONɑ 15.5 ± 1.0 μm, *n* = 21 (Wilcoxon ranksum *p* < 0.001).

Different types of regularly placed RGC types exhibited distinct density patterns across the retina, consistent with previous work on the distribution of RGCs in the mouse retina^16^. dRGCs were expected to mostly occur in the far periphery of the retina (outer marginal zone^12^) in a potentially random fashion. However, the analysis of displaced RGCs revealed cell-type specific patterns of cell bodies in the INL, which followed neither the general density distribution of all RGCs, nor those of the corresponding cell type (Fig. 7, right INL panel).

On average, 1264 ± 49 dRGCs were quantified per retina (*n* = 4). This represents an amount of 2.4 ± 0.2% of all RGCs. Cell bodies of dRGCs were accumulated in the retinal periphery, but a large fraction was also observed in more central parts of the retina, specifically in a region above the optic disc and in the dorso-temporal area (Fig. 7D-F). 154 ± 15 displaced M1 ipRGCs were counted per retina (*n* = 5). This represents 16.1 ± 1.9% of all M1 cells. Displaced M1 cell bodies were primarily clustered in a streak-like region above the optic disc (Fig. 7J-L). In sharp contrast, M2 cell bodies were rarely found to be displaced. On average, only 5 ± 4 displaced M2 ipRGCs were counted per retina (*n* = 5; Fig. 7P-R).

For this large-scale analysis of cell body positions across tile scans, we had to rely on epifluorescence imaging (see Methods), unlike the confocal scans for the SMI-32 tracings above (Fig. 4). Due to the associated limitations in optical resolution, it was not possible to reliably quantify the number and distribution of displaced sONɑ RGCs. They were positive for SMI-32 and calbindin, like their regularly placed counterparts, but the cell bodies were smaller (Fig. 3G), the staining intensities were relatively low, and often superimposed by intense fluorescence of horizontal cells. Thus, we relied on the combination of RBPMS, melanopsin, and osteopontin to analyze the population of displaced ɑRGCs without any differentiation of the four types (206 ± 35 displaced ɑRGCs per retina, *n* = 4). The distribution of displaced ɑRGCs resembled the distribution of sONɑ RGCs in the GCL only to a certain extent (Fig. 7V-X). Similar to regularly placed sONɑ RGCs (Fig. 7S-U), the analysis of displaced ɑRGCs (7.5 ± 1.1% of the total population of ɑRGCs) revealed a density peak region located in the dorso-temporal retina. However, the density peak of displaced ɑRGCs was located closer to the peripheral edge of the retina than the peak region of sONɑ RGCs in the GCL.

## Discussion

In this study, we introduced osteopontin as a marker for alpha RGCs and M2 ipRGCs which allowed us to analyze the type-specific distribution and spatial arrangement of dRGCs across complete retinas in direct relationship with their partners in the GCL. We show that the distribution of dRGCs does not follow the overall density distribution of cells in the GCL, in line with results from rat retinas^52^. Furthermore, we found that a displacement of cell bodies is type dependent, and we provide anatomical as well as electrophysiological evidence that dRGCs complete the tiling pattern of regular RGCs to form mosaics.

### Ganglion cell density gradients across the mouse retina

Originally, the general density distribution of mouse RGCs was described to be rather uniform, with a slight central-to-peripheral gradient^12,53^. With ongoing methodological progress regarding the use of molecular cell markers and functional analyses for cell type classification, it was shown that complex patterns of density gradients exist^16^. Here, we provide further evidence for distinct density distributions of all RGCs and those of individual types. In line with previous results from mouse and rat retinas^13,54^, the overall RGC density showed a steep increase from low density in the far dorsal retina to highest densities in a region above the optic disc and generally high densities in ventral retina. The density increase is roughly aligned with the transition zone of photoreceptor opsin expression (data not shown) and should therefore correspond to the horizon in the visual environment of the animal^55,56^.

The distribution patterns we observed for regular sONɑ and M1 ipRGCs also resembled previously reported results^11,45,57^, where it was shown that the dorso-temporal density peak of sONɑ RGCs aligns with the binocular region of the visual field of the mouse. However, the density of M2 ipRGCs appeared different from previous reports^20,45^, i.e. about two times lower in our study. This discrepancy is likely caused by the analysis of whole retinas here versus quantification of much smaller tissue regions in other studies. Extrapolation of values from the M2 ipRGC density from the dorsal peak regions (Fig. 7O) for a whole retina, for instance, would yield numbers that matched previously published results. It is rather unlikely that we missed a large fraction of cells in our quantification due to potentially weak melanopsin immunostaining, because weakly melanopsin-positive sONɑ RGCs were frequently observed in our samples without using further enhancement techniques^32^. Therefore, the observation of melanopsin-positive sONɑ RGCs served as an intrinsic control for optimal staining intensities.

### Density gradients and spatial arrangement of displaced ganglion cells

Displaced ganglion cells have been shown to exist in various vertebrate species, such as fish, birds, and mammals including human retina ^[e.g. 12,13,52,54,58–65^. However, it remained unclear whether displaced RGCs represent independent cell types, an odd part of the population of regular types, or just futile remnants of disordered neuronal development. Hallmark features of regular RGC types within local retinal regions are their uniform dendritic morphologies and the corresponding formation of independent mosaics, as well as uniform light response properties^66^. While the existence of dRGCs was known, neither their spatial arrangement relative to regularly placed RGCs nor their light response properties have been explored.

Our data now shows that, at least for two identified RGC types of the mouse retina, dRGCs are part of the population of their regularly placed counterparts and that they are arranged as proper cellular components of the corresponding mosaic. The dendritic structure of dRGCs and the dendritic overlap with their neighbors was not obviously different from regularly placed RGCs. Interestingly, the frequency of their occurrence was not dictated by the densities of their regular counterparts, assuming that higher numbers of regular cells would increase the chance of a displacement to happen. Previous studies suggested that dRGCs in the mammalian retina are misplaced due to ontogenetic aberrations^34^. While the cause of displacement remains elusive, it is unlikely to happen randomly, as the patterns of displacement shown here occurred in a type-specific manner, consistently across multiple samples. Nevertheless, cell-type-specific gene expression levels during development may play a role in the formation of dRGC patterns. It was shown that the extracellular matrix protein glycogen synthase kinase 3 (GSK3) contributes to the spatial organization of RGCs^67^. The lack of one GSK3 allele led to increased numbers of dRGCs, but not by overproduction of cells as total RGC numbers were stable. It remains a matter of speculation that sONɑ and M1 RGCs may express lower levels of GSK3 during development, and that, therefore, their migration to the GCL is more frequently disturbed than in M2 RGCs, for instance. M2 cells were rarely found to be displaced, further supporting the notion of a cell-type dependent mechanism.

It has been shown in murine retinas that dRGCs are a highly diverse group that comprises cells with a large morphological variety^33,34^. The displaced alpha RGCs together with the displaced M1 and M2 ipRGCs added up to only ~ 30% of all displaced RGCs which we observed (with M2 ipRGCs accounting for < 1%). Morphological changes across cell types according to regional variations and density gradients^16^ could have led to a slight overestimation of dRGC variety in previous studies. Nevertheless, one would still expect to find displaced cell bodies from many other RGC types beyond the three types investigated here. Whether all mouse dRGCs follow the rules which we found to apply to this group of cells remains to be elucidated.

### Do displaced ganglion cells serve special functional roles?

Our data shows that dRGCs complete the dendritic and corresponding functional receptive field mosaics of their regularly placed partners and that the basic light response properties do not differ between dRGCs and other cells of that mosaic. Therefore, we conclude that they belong to the same type and serve the same functional roles. However, the type-specific patterns of displacement, and the formation of a streak-like area by displaced M1 ipRGCs that is not apparent in their regularly placed partners, for instance, fuel the idea that a special role of mouse dRGCs remains to be discovered.

In the avian retina, a subpopulation of dRGCs is indeed thought to be an independent cell type^68^. dRGCs project exclusively to the nucleus of the basal optic root (nBOR) and are therefore thought to be responsible for the optokinetic nystagmus and involved in retinal image stabilization^35–37,69^. The mammalian homolog of the avian nBOR is the medial terminal nucleus (MTN). It was shown that this region receives little or no input from dRGCs in rabbit^70^. However, an increased number of dRGCs in genetically modified mice was accompanied by higher numbers of RGC axon terminals in MTN^67^. Therefore, results regarding the innervation of the mammalian MTN from dRGCs remain inconclusive and require further studies to allow insights into potential roles of displaced RGC types in eye movements.

It has been shown that displaced ipRGCs are accumulated in an area above the optic disc in mouse retinas^71^. Here, we provide evidence that this phenomenon occurs due to a streak-like density peak of displaced M1 ipRGCs. A conclusive classification of M1 ipRGCs remains a challenge, as they may comprise subtypes, which exhibit functional differences, different levels of melanopsin, distinct gene expression patterns and project to different target areas^50,72–75^. It is tempting to speculate that a specific M1 subtype is responsible for the enrichment of dRGCs in this streak-like area considering that ipRGC subtypes may form independent mosaics^31^. However, this remains a subject of future studies once reliable tools are available to specifically distinguish putative M1 subtypes^76^.

How could dRGCs potentially benefit from a targeted, cell-type specific displacement of their cell bodies? Changes in the common synaptic circuitry can now be ruled out, as all dendritic trees stratify at the same level and would presumably interact with the same synaptic partners. However, direct synaptic inputs to the cell bodies of dRGCs may play a key role, formed by presynaptic cells stratifying in the most distal IPL layer. An example of such a circuitry at the IPL-to-INL border is the perinuclear nests which are formed by dopaminergic amacrine cells around the cell bodies of postsynaptic AII amacrine cells^77–79^. Dendro-somatic synaptic interactions were also shown to occur at regular ganglion cell bodies with great functional impact^80^ and could represent a circuit motif for amacrine-dRGCs interactions in the INL as well. Furthermore, it is known from a subset of dRGCs to express distinct voltage-gated calcium channel subunits in murine retinas, which were not found in regular RGCs, suggesting that those dRGCs may have distinct functional properties^81^.

Thus, our data suggests that mouse dRGCs primarily fulfill the same functional role like their regular counterparts. However, additional functional features may be discovered by a detailed study of physiological and genetic properties of dRGCs in direct association with their regularly placed partners.

## Electronic supplementary material

Below is the link to the electronic supplementary material.

Supplementary Material 1

## Data Availability

Data are available from the corresponding author upon reasonable request.

## References

[1] Baden, T. et al. The functional diversity of retinal ganglion cells in the mouse. *Nature* **529**, 345–350 (2016). 26735013 10.1038/nature16468 PMC4724341

[2] Tran, N. M. et al. Single-Cell profiles of retinal ganglion cells differing in resilience to injury reveal neuroprotective genes. *Neuron* **104**, 1039–1055e12 (2019). 31784286 10.1016/j.neuron.2019.11.006 PMC6923571

[3] Doi, M., Uji, Y. & Yamamura, H. Morphological classification of retinal ganglion cells in mice. *J. Comp. Neurol.* **356**, 368–386 (1995). 7543910 10.1002/cne.903560305

[4] Sun, W., Li, N. & He, S. Large-scale morphological survey of mouse retinal ganglion cells. *J. Comp. Neurol.* **451**, 115–126 (2002). 12209831 10.1002/cne.10323

[5] Wässle, H. & Riemann, H. J. The mosaic of nerve cells in the mammalian retina. 24 (1978). 10.1098/rspb.1978.0026 26058

[6] Wässle, H., Peichl, L. & Boycott, B. B. Dendritic territories of Cat retinal ganglion cells. *Nature* **292**, 344–345 (1981). 7254331 10.1038/292344a0

[7] Stone, J. A quantitative analysis of the distribution of ganglion cells in the Cat’s retina. *J. Comp. Neurol.* **124**, 337–352 (1965). 4955540 10.1002/cne.901240305

[8] Oyster, C. W., Takahashi, E. S. & Hurst, D. C. Density, Soma size, and regional distribution of rabbit retinal ganglion cells. *J. Neurosci. Off J. Soc. Neurosci.* **1**, 1331–1346 (1981). 10.1523/JNEUROSCI.01-12-01331.1981 PMC6564131 7320749

[9] Curcio, C. A. & Allen, K. A. Topography of ganglion cells in human retina. *J. Comp. Neurol.* **300**, 5–25 (1990). 2229487 10.1002/cne.903000103

[10] Collin, S. P. A web-based archive for topographic maps of retinal cell distribution in vertebrates. *Clin. Exp. Optom.* **91**, 85–95 (2008). 18045254 10.1111/j.1444-0938.2007.00228.x

[11] Bleckert, A., Schwartz, G. W., Turner, M. H., Rieke, F. & Wong, R. O. L. Visual space is represented by nonmatching topographies of distinct mouse retinal ganglion cell types. *Curr. Biol.* **24**, 310–315 (2014). 24440397 10.1016/j.cub.2013.12.020 PMC3990865

[12] Dräger, U. C. & Olsen, J. F. *Ganglion Cell. Distribution Retina Mouse* **20**, 9 (1981). 6162818

[13] Salinas-Navarro, M. et al. Retinal ganglion cell population in adult albino and pigmented mice: A computerized analysis of the entire population and its Spatial distribution. *Vis. Res.* **49**, 637–647 (2009). 19948111 10.1016/j.visres.2009.01.010

[14] Zhang, Y., Kim, I. J., Sanes, J. R. & Meister, M. The most numerous ganglion cell type of the mouse retina is a selective feature detector. *Proc. Natl. Acad. Sci. U S A*. **109**, E2391–E2398 (2012). 22891316 10.1073/pnas.1211547109 PMC3437843

[15] Rousso, D. L. et al. Two pairs of ON and OFF retinal ganglion cells are defined by intersectional patterns of transcription factor expression. *Cell. Rep.* **15**, 1930–1944 (2016). 27210758 10.1016/j.celrep.2016.04.069 PMC4889540

[16] Heukamp, A. S., Warwick, R. A. & Rivlin-Etzion, M. Topographic variations in retinal encoding of visual space. *Annu. Rev. Vis. Sci.* **6**, 237–259 (2020). 32320630 10.1146/annurev-vision-121219-081831

[17] Provencio, I. et al. A novel human Opsin in the inner retina. *J. Neurosci.* **20**, 600–605 (2000). 10632589 10.1523/JNEUROSCI.20-02-00600.2000 PMC6772411

[18] Berson, D. M., Dunn, F. A. & Takao, M. Phototransduction by retinal ganglion cells that set the circadian clock. **295**, 5 (2002). 10.1126/science.1067262 11834835

[19] Hattar, S., Liao, H. W., Takao, M., Berson, D. M. & Yau, K. W. Melanopsin-Containing retinal ganglion cells: architecture, projections, and intrinsic photosensitivity. *Science* **295**, 1065–1070 (2002). 11834834 10.1126/science.1069609 PMC2885915

[20] Berson, D. M., Castrucci, A. M. & Provencio, I. Morphology and mosaics of melanopsin?expressing retinal ganglion cell types in mice. *J. Comp. Neurol.* **18** (2010). 10.1002/cne.22381 PMC2895505 20503419

[21] Nasir-Ahmad, S., Lee, S. C. S., Martin, P. R. & Grünert, U. Melanopsin-expressing ganglion cells in human retina: morphology, distribution, and synaptic connections. *J. Comp. Neurol.* **527**, 312–327 (2019). 28097654 10.1002/cne.24176

[22] Haverkamp, S., Mietsch, M. & Briggman, K. L. Developmental errors in the common marmoset retina. *Front. Neuroanat.* **16**, (2022). 10.3389/fnana.2022.1000693 PMC9531312 36204677

[23] Fitzpatrick, M. J., Krizan, J., Hsiang, J. C. & Shen, N. Kerschensteiner, D. A pupillary contrast response in mice and humans: neural mechanisms and visual functions. *Neuron* **112**, 2404–2422e9 (2024). 38697114 10.1016/j.neuron.2024.04.012 PMC11257825

[24] Gao, J. et al. Differential effects of experimental Glaucoma on intrinsically photosensitive retinal ganglion cells in mice. *J. Comp. Neurol.* **530**, 1494 (2022). 34958682 10.1002/cne.25293 PMC9010357

[25] Aranda, M. L. & Schmidt, T. M. Diversity of intrinsically photosensitive retinal ganglion cells: circuits and functions. *Cell. Mol. Life Sci. CMLS*. **78**, 889–907 (2021). 32965515 10.1007/s00018-020-03641-5 PMC8650628

[26] Ecker, J. L. Melanopsin-Expressing retinal Ganglion-Cell photoreceptors: cellular diversity and role in pattern vision. **12** (2010). 10.1016/j.neuron.2010.05.023 PMC2904318 20624591

[27] Quattrochi, L. E. et al. The M6 cell: A small-field bistratified photosensitive retinal ganglion cell. *J. Comp. Neurol.* **527**, 297–311 (2019). 30311650 10.1002/cne.24556 PMC6594700

[28] Schmidt, T. M., Chen, S. K. & Hattar, S. Intrinsically photosensitive retinal ganglion cells: many subtypes, diverse functions. *Trends Neurosci.* **34**, 572–580 (2011). 21816493 10.1016/j.tins.2011.07.001 PMC3200463

[29] Schmidt, T. M. et al. A role for melanopsin in alpha retinal ganglion cells and contrast detection. *Neuron* **82**, 781–788 (2014). 24853938 10.1016/j.neuron.2014.03.022 PMC4083763

[30] Stabio, M. E. et al. The M5 cell: A Color-Opponent intrinsically photosensitive retinal ganglion cell. *Neuron* **97**, 150–163e4 (2018). 29249284 10.1016/j.neuron.2017.11.030 PMC5757626

[31] Berry, M. H. et al. A melanopsin ganglion cell subtype forms a dorsal retinal mosaic projecting to the supraoptic nucleus. *Nat. Commun.* **14**, 1492 (2023). 36932080 10.1038/s41467-023-36955-6 PMC10023714

[32] Estevez, M. E. et al. Form and function of the M4 cell, an intrinsically photosensitive retinal ganglion cell type contributing to geniculocortical vision. *J. Neurosci.* **32**, 13608–13620 (2012). 23015450 10.1523/JNEUROSCI.1422-12.2012 PMC3474539

[33] Pang, J. J. & Wu, S. M. Morphology and immunoreactivity of retrogradely Double-Labeled ganglion cells in the mouse retina. *Investig Opthalmology Vis. Sci.* **52**, 4886 (2011). 10.1167/iovs.10-5921 PMC3175970 21482641

[34] Buhl, E. H. & Dann, J. F. Morphological diversity of displaced retinal ganglion cells in the rat: A lucifer yellow study. *J. Comp. Neurol.* **269**, 210–218 (1988). 3356810 10.1002/cne.902690206

[35] Simpson, J. I. The accessory optic system. *Annu. Rev. Neurosci.* **7**, 13–41 (1984). 6370078 10.1146/annurev.ne.07.030184.000305

[36] Dann, J. F. & Buhl, E. H. Retinal ganglion cells projecting to the accessory optic system in the rat. *J. Comp. Neurol.* **262**, 141–158 (1987). 3624547 10.1002/cne.902620111

[37] Cook, J. E. & Podugolnikova, T. A. Evidence for Spatial regularity among retinal ganglion cells that project to the accessory optic system in a frog, a reptile, a bird, and a mammal. *Vis. Neurosci.* **18**, 289–297 (2001). 11417803 10.1017/s0952523801182131

[38] Feng, G. et al. Imaging neuronal subsets in Transgenic mice expressing multiple spectral variants of GFP. *Neuron* **28**, 41–51 (2000). 11086982 10.1016/s0896-6273(00)00084-2

[39] Stabio, M. E. et al. A novel map of the mouse eye for orienting retinal topography in anatomical space. *J. Comp. Neurol.* **526**, 1749–1759 (2018). 29633277 10.1002/cne.24446 PMC5990451

[40] Rodieck, R. W. The density recovery profile: A method for the analysis of points in the plane applicable to retinal studies. *Vis. Neurosci.* **6**, 95–111 (1991). 2049333 10.1017/s095252380001049x

[41] Sterratt, D. C., Lyngholm, D., Willshaw, D. J. & Thompson, I. D. Standard anatomical and visual space for the mouse retina: computational reconstruction and transformation of flattened retinae with the retistruct package. *PLoS Comput. Biol.* **9**, e1002921 (2013). 23468609 10.1371/journal.pcbi.1002921 PMC3585388

[42] Schmucker, C. & Schaeffel, F. A paraxial schematic eye model for the growing C57BL/6 mouse. *Vis. Res.* **44**, 1857–1867 (2004). 15145680 10.1016/j.visres.2004.03.011

[43] Field, G. D. et al. Spatial properties and functional organization of small bistratified ganglion cells in primate retina. *J. Neurosci.* **27**, 13261–13272 (2007). 18045920 10.1523/JNEUROSCI.3437-07.2007 PMC6673390

[44] Litke, A. M. et al. What does the eye tell the brain? Development of a system for the large-scale recording of retinal output activity. *IEEE Trans. Nucl. Sci.* **51**, 1434–1440 (2004).

[45] Hughes, S., Watson, T. S., Foster, R. G., Peirson, S. N. & Hankins, M. W. Nonuniform distribution and spectral tuning of photosensitive retinal ganglion cells of the mouse retina. *Curr. Biol.* **23**, 1696–1701 (2013). 23954426 10.1016/j.cub.2013.07.010 PMC3770899

[46] Duan, X. Subtype-Specific regeneration of retinal ganglion cells following axotomy: effects of osteopontin and mTOR. *Signaling* 22 (2015). 10.1016/j.neuron.2015.02.017 PMC4391013 25754821

[47] Krieger, B., Qiao, M., Rousso, D. L., Sanes, J. R. & Meister, M. Four alpha ganglion cell types in mouse retina: function, structure, and molecular signatures. *PLOS ONE*. **12**, e0180091 (2017). 28753612 10.1371/journal.pone.0180091 PMC5533432

[48] Straznicky, C., Vickers, J. C., Gábriel, R. & Costa, M. A neurofilament protein antibody selectively labels a large ganglion cell type in the human retina. *Brain Res.* **582**, 123–128 (1992). 1498675 10.1016/0006-8993(92)90325-4

[49] Rodriguez, A. R., de Sevilla Müller, L. P. & Brecha, N. C. The RNA binding protein RBPMS is a selective marker of ganglion cells in the mammalian retina. *J. Comp. Neurol.* **522**, 1411–1443 (2014). 24318667 10.1002/cne.23521 PMC3959221

[50] Schmidt, T. M. & Kofuji, P. Functional and morphological differences among intrinsically photosensitive retinal ganglion cells. **7** (2009). 10.1523/JNEUROSCI.4117-08.2009 PMC2752349 19144848

[51] Neumann, S., Hüser, L., Ondreka, K., Auler, N. & Haverkamp, S. Cell type-specific bipolar cell input to ganglion cells in the mouse retina. *Neuroscience* **316**, 420–432 (2016). 26751712 10.1016/j.neuroscience.2015.12.051

[52] Nadal-Nicolás, F. M. et al. Displaced retinal ganglion cells in albino and pigmented rats. *Front. Neuroanat.* **8**, 99 (2014). 25339868 10.3389/fnana.2014.00099 PMC4186482

[53] Jeon, C. J., Strettoi, E. & Masland, R. H. The major cell populations of the mouse retina. *J. Neurosci.* **18**, 8936–8946 (1998). 9786999 10.1523/JNEUROSCI.18-21-08936.1998 PMC6793518

[54] Salinas-Navarro, M. et al. A computerized analysis of the entire retinal ganglion cell population and its Spatial distribution in adult rats. *Vis. Res.* **49**, 115–126 (2009). 18952118 10.1016/j.visres.2008.09.029

[55] Baden, T. et al. A Tale of two retinal domains: Near-Optimal sampling of achromatic contrasts in natural scenes through asymmetric photoreceptor distribution. *Neuron* **80**, 1206–1217 (2013). 24314730 10.1016/j.neuron.2013.09.030

[56] Nadal-Nicolás, F. M. et al. True S-cones are concentrated in the ventral mouse retina and wired for color detection in the upper visual field. *eLife* **9**, e56840 (2020). 10.7554/eLife.56840 PMC7308094 32463363

[57] Johnson, K. P. et al. Cell-type-specific binocular vision guides predation in mice. *Neuron* **109**, 1527–1539e4 (2021). 33784498 10.1016/j.neuron.2021.03.010 PMC8112612

[58] Dacey, D. M. et al. Melanopsin-expressing ganglion cells in primate retina signal colour and irradiance and project to the LGN. *Nature* **433**, 749–754 (2005). 15716953 10.1038/nature03387

[59] Chandra, A. J., Lee, S. C. S. & Grünert, U. Melanopsin and Calbindin immunoreactivity in the inner retina of humans and marmosets. *Vis. Neurosci.* **36**, E009 (2019). 31581958 10.1017/S0952523819000087

[60] Hoshi, H. & Sato, F. The morphological characterization of orientation-biased displaced large-field ganglion cells in the central part of goldfish retina. *J. Comp. Neurol.* **526**, 243–261 (2018). 28921532 10.1002/cne.24331

[61] Mey, J. & Johann, V. Dendrite development and target innervation of displaced retinal ganglion cells of the chick (Gallus gallus). *Int. J. Dev. Neurosci. Off J. Int. Soc. Dev. Neurosci.* **19**, 517–531 (2001). 10.1016/s0736-5748(01)00030-2 11470381

[62] Bunt, A. H. & Minckler, D. S. Displaced ganglion cells in the retina of the monkey. *Invest. Ophthalmol. Vis. Sci.* **16**, 95–98 (1977). 401780

[63] Stell, W. K. & Witkovsky, P. Retinal structure in the smooth dogfish, Mustelus Canis: general description and light microscopy of giant ganglion cells. *J. Comp. Neurol.* **148**, 1–31 (1973). 4121526 10.1002/cne.901480102

[64] Tachibana, M. Displaced ganglion cells in carp retina revealed by the horseradish peroxidase technique. *Neurosci. Lett.* **9**, 153–157 (1978). 19605210 10.1016/0304-3940(78)90064-2

[65] Robson, J. A. & Holländer, H. Displaced ganglion cells in the rabbit retina. *Invest. Ophthalmol. Vis. Sci.* **25**, 1376–1381 (1984). 6511223

[66] Vlasits, A. L., Euler, T. & Franke, K. Function first: classifying cell types and circuits of the retina. *Curr. Opin. Neurobiol.* **56**, 8–15 (2019). 30447507 10.1016/j.conb.2018.10.011

[67] Kisseleff, E. et al. Glycogen Synthase Kinase 3 Regulates the Genesis of Displaced Retinal Ganglion Cells3. *eNeuro* 8, ENEURO.0171-21.2021 (2021). 10.1523/ENEURO.0171-21.2021 PMC8496207 34518365

[68] Haverkamp, S., Albert, L., Balaji, V., Němec, P. & Dedek, K. Expression of cell markers and transcription factors in the avian retina compared with that in the marmoset retina. *J. Comp. Neurol.* **529**, 3171–3193 (2021). 33834511 10.1002/cne.25154

[69] Fite, K. V., Brecha, N., Karten, H. J. & Hunt, S. P. Displaced ganglion cells and the accessory optic system of pigeon. *J. Comp. Neurol.* **195**, 279–288 (1981). 7251927 10.1002/cne.901950208

[70] Oyster, C. W., Simpson, J. I., Takahashi, E. S. & Soodak, R. E. Retinal ganglion cells projecting to the rabbit accessory optic system. *J. Comp. Neurol.* **190**, 49–61 (1980). 7381054 10.1002/cne.901900105

[71] Valiente-Soriano, F. J. et al. Distribution of melanopsin positive neurons in pigmented and albino mice: evidence for melanopsin interneurons in the mouse retina. *Front. Neuroanat.* **8**, 131 (2014). 25477787 10.3389/fnana.2014.00131 PMC4238377

[72] Hattar, S. et al. Central projections of melanopsin-expressing retinal ganglion cells in the mouse. *J. Comp. Neurol.* **497**, 326–349 (2006). 16736474 10.1002/cne.20970 PMC2885916

[73] Jain, V., Ravindran, E. & Dhingra, N. K. Differential expression of Brn3 transcription factors in intrinsically photosensitive retinal ganglion cells in mouse. *J. Comp. Neurol.* **520**, 742–755 (2012). 21935940 10.1002/cne.22765

[74] Li, J. Y. & Schmidt, T. M. Divergent projection patterns of M1 IpRGC subtypes. *J. Comp. Neurol.* **526**, 2010–2018 (2018). 29888785 10.1002/cne.24469 PMC6158116

[75] Emanuel, A. J., Kapur, K. & Do, M. T. H. Biophysical variation within the M1 type of ganglion cell photoreceptor. *Cell. Rep.* **21**, 1048–1062 (2017). 29069587 10.1016/j.celrep.2017.09.095 PMC5675019

[76] Laboissonniere, L. A. et al. Molecular signatures of retinal ganglion cells revealed through single cell profiling. *Sci. Rep.* **9**, 15778 (2019). 31673015 10.1038/s41598-019-52215-4 PMC6823391

[77] Voigt, T. & Wassle, H. Dopaminergic innervation of A II Amacrine cells in mammalian retina. *J. Neurosci.* **7**, 4115–4128 (1987). 2891802 10.1523/JNEUROSCI.07-12-04115.1987 PMC6569108

[78] Casini, G., Rickman, D. W. & Brecha, N. C. AII Amacrine cell population in the rabbit retina: identification by parvalbumin immunoreactivity. *J. Comp. Neurol.* **356**, 132–142 (1995). 7629307 10.1002/cne.903560109

[79] Contini, M. & Raviola, E. GABAergic synapses made by a retinal dopaminergic neuron. *Proc. Natl. Acad. Sci.* **100**, 1358–1363 (2003). 12547914 10.1073/pnas.0337681100 PMC298777

[80] Grimes, W. N. et al. Dendro-somatic synaptic inputs to ganglion cells contradict receptive field and connectivity conventions in the mammalian retina. *Curr. Biol.* **32**, 315–328e4 (2022). 34822767 10.1016/j.cub.2021.11.005 PMC8792273

[81] De Sevilla Müller, L. P., Liu, J., Solomon, A., Rodriguez, A. & Brecha, N. C. Expression of Voltage-Gated calcium channel Α2δ4 subunits in the mouse and rat retina. *J. Comp. Neurol.* **521**, 2486–2501 (2013). 23296739 10.1002/cne.23294 PMC3748150

