# Supplementary material for: Spatial distribution and functional integration of displaced retinal ganglion cells
Source: Sci Rep. 2025 Feb 28;15:7123. doi: 10.1038/s41598-025-91045-5 (PMC11868576; doi:10.1038/s41598-025-91045-5)
Supplement: Supplementary file 1 — Supplementary Material 1 [file 41598_2025_91045_MOESM1_ESM.pdf]

Supplementary Information, related to Figure 4

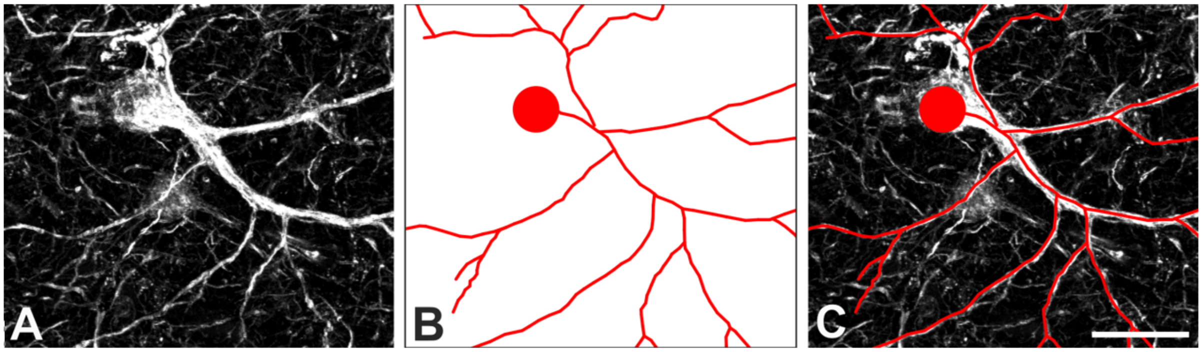

Figure S1: SMI-32 labeling and tracing of a displaced M4/sONα cell.

A: Maximum intensity projection of a confocal image stack of whole-mounted retina labeled against SMI-32. The projection includes parts of the inner nuclear layer and most of the inner plexiform layer. **B:** Tracing of the cell body and primary dendrites of the displaced M4/sONα cell from Fig.4A-C. **C:** Overlay. Scale bar: 25 µm.
